# Extraperitoneal Robot-Assisted Laparoscopic Radical Prostatectomy in Continuous Spinal Anesthesia: A New Approach to an Established Technique

**DOI:** 10.3390/medicina60121973

**Published:** 2024-12-01

**Authors:** Simone Morselli, Laura Zavatti, Riccardo Ferrari, Lorenzo Gatti, Salvatore Micali, Salvatore Rabito, Luca Cindolo, Giovanni Ferrari

**Affiliations:** 1 Department of Urology, C.Ur.E.—Centro Urologico Europeo, Hesperia Hospital, 41125 Modena, Italy; rferrari@hesperia.it (R.F.); lgatti@hesperia.it (L.G.); srabito@hesperia.it (S.R.); lucacindolo@virgilio.it (L.C.); gferrari@hesperia.it (G.F.); 2 Department of Clinical and Experimental Medicine, University of Florence, 50121 Florence, Italy; 3 Department of Anesthesiology, Hesperia Hospital, 41125 Modena, Italy; lzavatti@hesperia.it; 4 Department of Urology, University of Modena and Reggio Emilia, 41121 Modena, Italy; salvatore.micali@unimore.it

**Keywords:** RALP, PCa, prostate cancer, radical prostatectomy, extraperitoneal, continuous spinal anesthesia, anesthesia, continuous spinal

## Abstract

*Background and Objectives:* To prove the feasibility of continuous spinal extraperitoneal robot-assisted laparoscopic radical prostatectomy (cseRALP) in order to expand the pool of eligible patients. *Materials and Methods*: According to IDEAL guidelines, a consecutive cohort of patients who underwent cseRALP was enrolled. Pre-, intra-, and post-operative data were collected, with particular focus on safety and oncological outcomes. *Results*: A total of three patients underwent this technique, with no intra- or post-operative medical complications. Only a grade 1 Clavien–Dindo complication was reported, small urinary leakage treated with an indwelling catheter. Oncological and functional results at month 3 were satisfactory, with no recurrence and no stress incontinence. *Conclusions*: cseRALP seems to be feasible and safe; further trials are mandatory.

## 1. Introduction

According to European Association of Urology (EAU) guidelines, robot-assisted laparoscopic radical prostatectomy (RALP) is a well-established technique for the treatment of localized Prostate Cancer (PCa), and it has undergone continuous progress over time [1,2]. Its main limitations are the transperitoneal approach, which represents contraindications in patients with prior extensive abdominal surgery, and the steep Trendelenburg position required during the surgery. To overcome these drawbacks, an extraperitoneal approach has been developed and, over time, it has been applied to robotic surgery [3]. Regardless of the surgical aspect, as the feasibility and the outcomes of extraperitoneal and transperitoneal RALP are proven to be comparable, the main difference involves medical or anesthesiological contraindications to RALP; in fact, the Trendelenburg position can be reduced in the extraperitoneal approach, providing theoretical benefits in morbidly obese patients and in cases of glaucoma, lung bullae, or severe chronic obstructive pulmonary disease [4,5,6]. Therefore, a further step to extend the range of patients who can benefit from extraperitoneal RALP was conceived at our center, through the development of an extraperitoneal RALP technique in continuous spinal anesthesia (cseRALP). The aim of this study is therefore to prove its feasibility and to present the first case series with this approach.

## 2. Materials and Methods

A prospective feasibility study on this technique was designed and applied at our center by a single surgeon, an expert in robotic and laparoscopic urological surgery, and by the referral anesthesiologist of the urological team. The study was designed according to the IDEAL guidelines and its various phases: the Idea (phase 1), the Development (phase 2a), the Exploration (phase 2b), the Assessment (phase 3), and the Long-term study (phase 4). Due to the experimental nature of the technique, we place this prospective study in Phase 2b, Exploration [7].

In detail, the Idea was developed through discussion, experience reporting, and a literature review of the possible available approaches to reduce the Trendelenburg angle. Considering our previous and large experience with laparoscopic radical prostatectomy and with the relative and absolute contraindications to RALP and its standard approach, we aimed to extend the robotic approach to as many patients as possible through an easily reproducible technique. Thus, the Development came with defining the technical aspects to be applied during surgery, in particular for patients’ positioning and details of the surgical and anesthesiologic technique, which will be subsequently reported. Finally, we began the Exploration phase, studying the outcomes and complications of this surgical technique in a series of patients, Strengthening the Reporting of Observational Studies in Epidemiology (STROBE) guidelines as reported in Supplementary File S1. Enrollment began in December 2021 and it is still ongoing. We included all patients referring to our center with PCa who had undergone the cseRALP surgical protocol. Their data were recorded in a prospective database of an observational study which was previously approved by the local Ethics Committee 350/2021/OSS/ESTMO.

### 2.1. Surgical Technique

**Figure 1:**
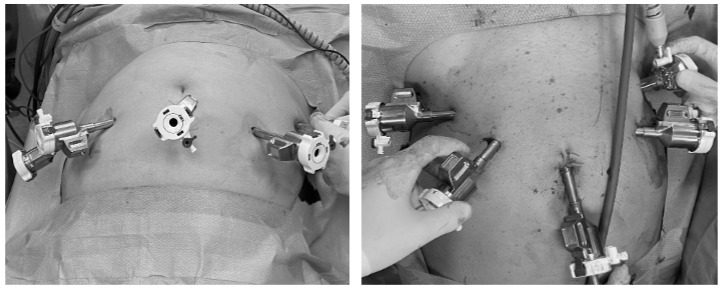
Trocar placement for continuous spinal extraperitoneal robot-assisted laparoscopic radical prostatectomy (**right side**) compared to standard robot-assisted laparoscopic prostatectomy (**left side**).

This technique consists of a modified eRALP, with the patient in a 10–12° Trendelenburg position. The port scheme is similar to the transperitoneal RALP, except for the position, which is slightly caudal, as shown in Figure 1, with the optical trocar of 8 mm placed below the umbilicus, contrary to standard RALP.

When the optic trocar is placed, the peritoneum is carefully spared and the extraperitoneal Retzius space is directly expanded with the finger or with a specific balloon filled with 500 cc of saline solution. Subsequently, under the field of view, the remaining three robotic 8 mm trocars and the 12 mm trocar for the bedside assistant surgeon are placed and the DaVinci^©^ Xi^©^ by Intuitive Surgical Inc. (Sunnyvale, CA, USA.) robot is docked. The surgery takes place as planned, with eventual Retzius space development. Then, defatting of the prostate is performed, with or without opening of the endopelvic fascia on the right or left side, according to the willingness to spare one or both the neurovascular bundles; in cases of bilateral nerve-sparing surgery, the endopelvic fascia is left untouched. The space between the prostate and bladder is carefully expanded, possibly sparing the bladder neck based on the eventual presence of a median prostate lobe. After that, the posterior space between the bladder and prostate is developed until the vas deferens and seminal vesicles are found and isolated. Therefore, the posterior space of the Denonvillier fascia is developed to the prostate apex and then laterally on an intra-, inter-, or extracapsular level, according to the possibility of nerve-sparing surgery. Consequently, the Santorini dorsal venous complex is incised, and eventually, a suture is placed to achieve a satisfying hemostasis. Thus, the prostate apex and the urethra are progressively isolated, until the urethra is incised and the radical prostatectomy is completed. The specimen is subsequently placed into an endobag. Posterior reconstruction with Rocco stitch is routinely performed, then a Van Velthoven urethral–bladder anastomosis with 2 semicontinuous 3-0 adsorbable barbed sutures is completed on a 20 Ch foley catheter. Attempts are made to make the anastomosis watertight with 100 cc of saline and a drain is put in place through the 12 mm trocar. The instruments are finally removed within view, together with the trocars, and the specimen is retrieved from the midline incision [8,9].

### 2.2. Aspects of Continuous Spinal Anesthesia

We perform continuous spinal anesthesia with a 25-gauge catheter introduced through a 21 Sprotte needle. Because the scalpel cuts a small section of skin, puncture is made easier with the Sprotte needle. The patient is generally positioned in a sitting or lateral position. The puncture site is identified through an accurate inspection of the thoracic and lumbar column, seeking the best approach site, considering the anesthetic level desired, generally T9-10 for eRALP. After preparing a sterile thoracic and lumbar field and injecting local anesthesia with Lidocaine 2% in the needle insertion site, a skin incision of 1 mm is performed. The Sprotte needle is inserted, seeking sufficient free flow of cerebrospinal fluid, and then the 25-gauge catheter is introduced for 3 to 4 cm after the needle tip. Subsequently, the catheter stylet is removed and, after checking the CSF flow again, the anesthetic drugs are injected. Before starting the surgery, the anesthetic level achieved is tested through a pinprick test. The patient maintains spontaneous breathing during the entire procedure with oxygen through a nasal cannula. The catheter is also used during the operation to administer drugs to maintain a good sedation level (SAS 2-3) and an optimal anesthetic level. In cases of pain not completely controlled by spinal anesthesia, it is possible to administer titrated IV fentanyl or ketamine. The catheter is removed upon discharge from the recovery room, before the patient’s transfer to the surgical ward.

The absolute contraindications for continuous spinal anesthesia are the same as single-shot techniques: lack of patient consensus, coagulopathy, local infection at the puncture site, and metastatic spinal disease.

### 2.3. Study Population and Inclusion and Exclusion Criteria

The study population included patients who had undergone prior prostate fusion biopsy after urological evaluation which found suspicion of PCa due to high PSA or suspect/positive digital rectal examination and subsequent multiparametric prostate Magnetic Resonance Imaging (mpMRI). According to ISUP grade, adequate staging was conducted as appropriate according to EAU guidelines [1,10]. The inclusion criterion was the willingness to undergo cseRALP. Exclusion criteria were contraindications to cseRALP, especially previous laparoscopic bilateral inguinal hernia repair.

### 2.4. Outcomes Definition and Follow-Up

According to the preliminary and explorative phase of this surgical technique, the main aspect was its safety, and the focus was on complications, reported according to the Clavien–Dindo Scale and divided into peri-operative (within 30 days of surgery) and post-operative (after 30 days) [11]. Regarding the outcomes, oncological outcomes such as positive surgical margins, post-operative PSA (between day 30 and day 40), and positive lymph nodes were recorded. Functional outcomes were assessed through objective measurements including the validated International Consultation on Incontinence Questionnaire Short Form (ICIQ-SF) and the number of daily pads. Post-operative hemoglobin, indwelling bladder catheter time in days, and pain assessed through the VAS scale were also recorded [12,13].

### 2.5. Statistical Analysis

A simple descriptive statistical analysis of categorical and continuous variables was conducted as appropriate, with the first reported as number and percentage, and the latter as median and interquartile range. Statistical analysis was carried out with SPSS 29.0 by IBM^©^ (Armonk, NY, USA).

### 2.6. Ethics and Inclusion Criteria

According to the explorative nature of the study, the collection and analysis of patients’ data were carried out after ethical appraisal to record patients’ characteristics, and their surgical data were obtained under the previously recalled study code (350/2021/OSS/ESTMO). For each patient, written informed consent was obtained for data collection. On the other hand, the absence of consent or the inability to collect them were the exclusion criteria. The study was thus conducted in accordance with the Declaration of Helsinki [14].

## 3. Results

A total of three patients have undergone this surgical and anesthesiologic approach during the time of the study since October 2023 due to anesthesiologic contraindications to standard RALP or surgical relative contraindications to the transperitoneal approach, which are listed in Table 1. A patient had close-angle glaucoma that was better managed with a low Trendelenburg angle.

During and after surgery, we reported no complications or unexpected issues due to the anesthesia technique chosen.

Regarding surgical complications, only a Clavien 1 complication was reported, namely small urine leakage from the anastomosis, which required a longer period of indwelling catheter use. No medical complications were reported in these patients, either during or after surgery. Median post-operative pain, measured through the VAS scale, was 0 (0–1), mostly reported during the first post-operative day but well controlled with painkillers. No patient required painkillers after day one.

When it comes to oncological outcomes, all patients had negative surgical margins. Pathological and functional results and post-operative PSA are reported in Table 2.

Indeed, none of our patients reported stress incontinence after cseRALP or any other long-term complication requiring further surgery.

## 4. Discussion

According to our results, cseRALP seems to be a valid and feasible technique, with a good safety profile. It certainly requires good coordination between the anesthetic and urological team, but it allows for further expansion of the indications to RALP to patients who are currently not eligible, due to surgical or medical comorbidities.

Nevertheless, in the literature, eRALP has already been proven to be a viable option in a surgeon’s range of options. In fact, in their study, Jacobs et al. compared standard transperitoneal RALP and eRALP, finding no differences in terms of surgical margins or oncological results. On the contrary, eRALP seemed to even reduce bowel complications and ileus, blood loss, and length of hospital stay [15]. Similar results were recently achieved in a systematic review and meta-analysis by Uy et al., which confirmed that eRALP guarantees comparable oncological and functional outcomes, while allowing for a faster operative time, decreased length of post-operative stay, and decreased rates of post-operative ileus and inguinal hernia [16].

The drawback of this approach is a smaller working space compared to transperitoneal RALP and thus a higher difficulty in port placement, which requires a much more experienced surgeon to approach this technique. Also, the difficulty in carrying out extended or superextended lymphadenectomy represents a considerable limitation [3].

Moreover, some authors have reported that CO2 diffusion in the blood is higher with eRALP, which represents a contraindication in cases of severe obstructive respiratory disease [17]. However, other authors have also reported that, despite this issue, the ventilatory pressures were statistically higher in standard RALP compared to eRALP, implying fewer ventilatory issues [18]. In our case series, with continuous spinal anesthesia and with careful monitoring of patients, we did not report any pneumological complications or difficulty due to CO_2_ reabsorption, which, together with a mild Trendelenburg position, could lead to an extended range of subjects who might benefit from this approach.

A limitation in the application of this technique is indeed previous surgical treatment of inguinal hernias, especially if video laparoscopic surgery, which can make the extraperitoneal space hard to obtain, with possible injuries to the bladder or epigastric and inguinal vessels, thus making a transperitoneal approach safer [3].

It has also been reported that eRALP can increase the risk of lymphoceles, as the lack of reabsorption of the lymph by the peritoneum may result in an increased incidence of post-operative lymphoceles, even though we did not experience any of them in our series, despite one of the patients undergoing a lymphadenectomy [16]. However, for the same reasons, bowel complications or ileus after surgery are less likely to occur, as our case series confirms, due to the impossibility of an eventual urine leak reaching the peritoneal cavity, thus causing gastrointestinal complications [3,16].

Indeed, the strength of our study lies in demonstrating the feasibility and safety of this technique, thus paving the way to further extend its application and confirm its validity. The main limitations are the small number of cases reported, the lack of a previous power analysis, and the experimental nature of this study. Nevertheless, we should also remember that, as a feasibility study, no prior power analysis can be carried out and that without a feasibility study, we cannot proceed to establish a full investigative protocol with or without a comparative group. Indeed, it is in our interests to develop such a study in the future.

## 5. Conclusions

According to our study, cseRALP appears to be both feasible and safe. Therefore, it will be our task to further prove its validity in the future in comparison to standard transperitoneal RALP.

## Figures and Tables

**Table 1:** Characteristics of patients undergoing continuous spinal extraperitoneal robot-assisted laparoscopic radical prostatectomy.

Patient Number	Age	PSA (ng/mL)	Contraindication to RALP	ISUP at Biopsy	Nerve Sparing	Global Operative Time (Minutes)	Day of Discharge	Day of Bladder Catheter Removal	Complications
1	63	5.3	Extensive previous abdominal surgery	2	Bilateral	120	3	8	No
2	73	6.8	Glaucoma and recently treated aortic aneurism	4	No	150	4	12	Small entity urine leakage
3	69	5.4	Extensive previous abdominal surgery	3	Monolateral	150	4	8	No

Legend: PSA: prostate-specific antigen; RALP: robot-assisted laparoscopic radical prostatectomy; ISUP: International Society of Urological Pathology.

**Table 2:** Detailed outcomes of oncological and functional results of continuous spinal extraperitoneal robot-assisted laparoscopic radical prostatectomy.

Patient Number	Post-Operative PSA (ng/mL)	ISUP at Pathology	TNM Staging	Surgical Margins (mm)	Number of Removed Nodes	ICIQ-SF at 3 Months	Number of Pad/Day	Further Surgical Operations
1	0.01	1	pT2cNxR0	0	NA	0	0	No
2	0.01	2	pT2aN0R0	0	19	1	0	No
3	0.01	2	pT2cNxR0	0	NA	0	0	No

Legend: PSA: prostate-specific antigen; RALP: robot-assisted laparoscopic radical prostatectomy; ISUP: International Society of Urological Pathology; TNM: Tumor Node Metastasis; ICIQ-SF: International Consultation on Incontinence Questionnaire—Urinary Incontinence Short Form; NA: not applicable.

## Data Availability

Data cannot be shared due to privacy issues, but they can be partly shared upon reasonable request.

## Supplementary Materials

The following supporting information can be downloaded at: https://www.mdpi.com/article/10.3390/medicina60121973/s1, Supplementary File S1: STROBE Statement—checklist of items that should be included in reports of observational studies [19].
